# Supplementary material for: Antiferromagnetic domain wall as spin wave polarizer and retarder
Source: Nat Commun. 2017 Aug 2;8:178. doi: 10.1038/s41467-017-00265-5 (PMC5541015; doi:10.1038/s41467-017-00265-5)
Supplement: Supplementary file 1 — Supplementary Information [file 41467_2017_265_MOESM1_ESM.pdf]

File Name: Supplementary Information
Description: Supplementary Figures, Supplementary Notes and Supplementary References

File Name: Peer Review File
Description:

File Name: Supplementary Movie
Description: Supplementary Movie 1.swf is a movie shows that a prepared spin spiral structure relaxes back to the domain wall structure in micromagnetic simuations for $tilde{D}=3.0\times 10^{-3} A$ and the free boundary condition. The basic micromagnetic simulation enviroment is the same as described in the section "Method" in the main text, but with damping rate increased to $alpha=0.02$ for faster relaxation.

In upper pannel, the magnetization component $m_1^{x/y/z$ is indicated by black/blue/red dots and line. The dots are from magnetic simulaltions, and the lines are from the standard Walker profile. In lower pannel, the red (blue) arrows are for the magnetization in sublattice 1/2.

Initially, the spin spiral state is prepared (and stablized) by turning on the DM boudnary condition. After changing the DM boundary condition into the free boundary condition, the spin spiral returns to the domain wall.

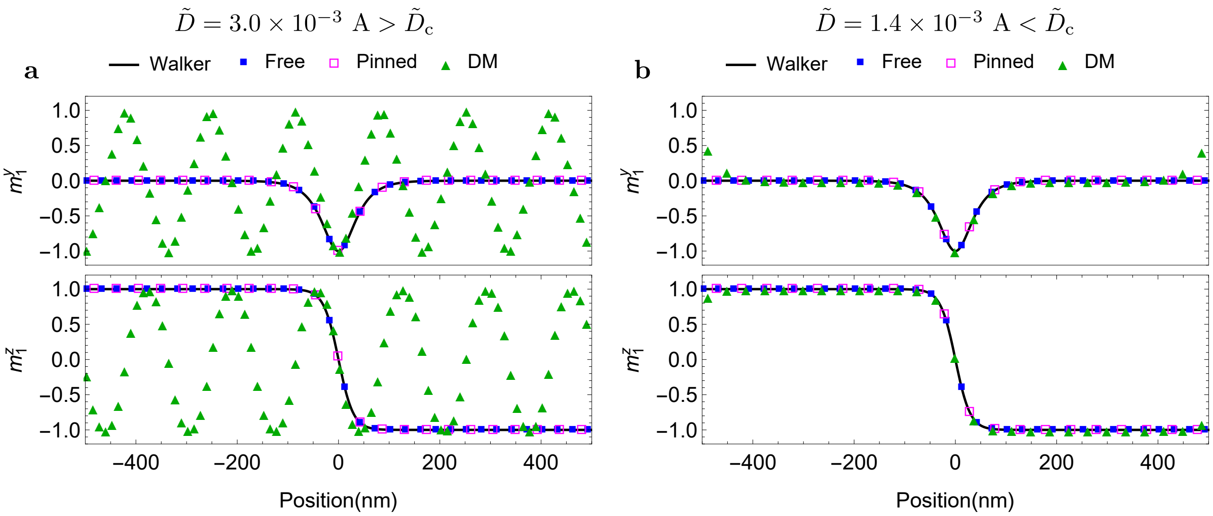

Supplementary Figure 1. **Micromagnetic simulations of static magnetic profiles with different boundary conditions**. The DMI strength is **a.** $\tilde{D} = 3.0 \times 10^{-3}$ A $> \tilde{D}_c$ (the value used in the main text), domain wall configuration is stable for free and pinned boundary condition, and spin spiral is stable for DM boundary condition. **b.** $\tilde{D} = 1.4 \times 10^{-3}$ A $< \tilde{D}_c$, domain wall configuration is always stable for all three types of boundary conditions. In both cases, the theoretical curves are the standard Walker profile.

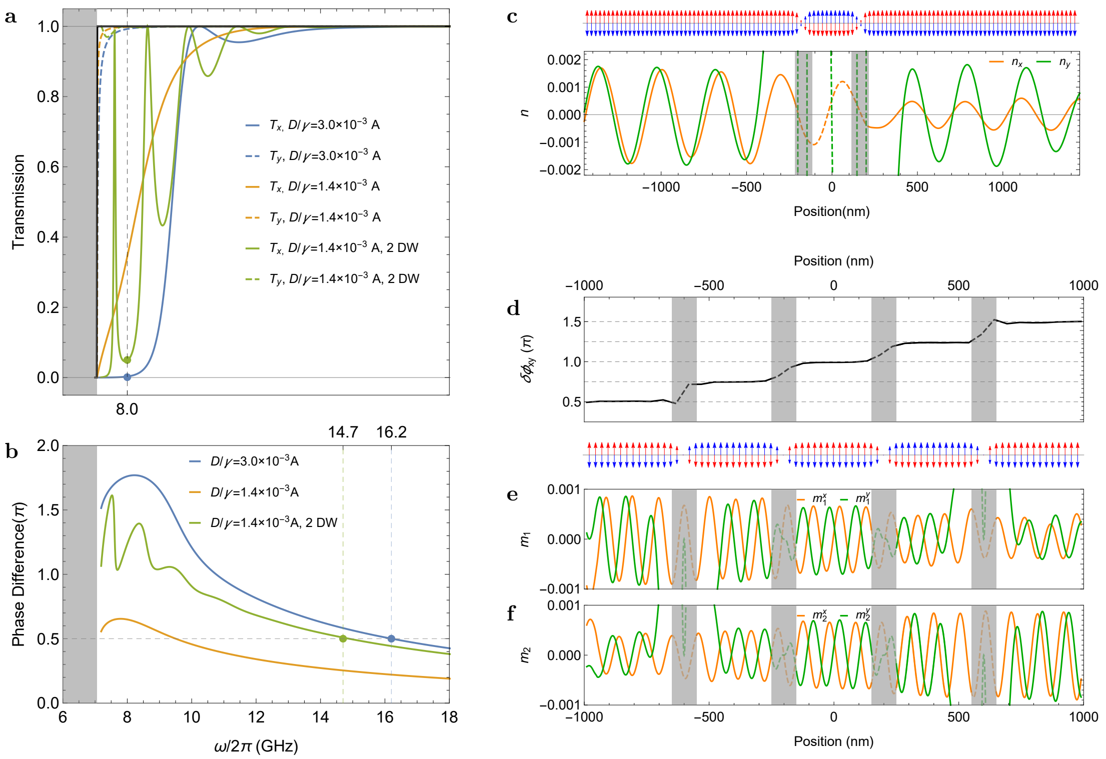

Supplementary Figure 2. **Theoretical calculations and micromagnetic simulations of the polarizing and retarding effects at $\tilde{D} = 1.4 \times 10^{-3}$ A $< \tilde{D}_c$. a-b.** The scattering behaviors across one or two antiferromagnetic domain walls for two different D values. **a.** The transmission probabilities for the x- and y-polarized spin wave modes. **b.** The relative phase delays between the x- and y-polarized modes. The double-domain-wall (2 DW) has a separation of 335 nm. All curves are calculated by using the Green function technique. **c.** Micromagnetic simulation of the polarizing effect with a double-domain-wall structure at a working frequency of 8.0 GHz. Circular spin waves are injected from the left side, and the transmitted waves are preferentially polarized along y. **d-f.** Micromagnetic simulation of the retarding effect with 4 consecutive domain walls at a working frequency of 14.7 GHz. Right-circular spin waves are injected from the left side. **d.** The relative phase delay between the x- and y-polarizations shows the stepwise behavior (upper panel) when passing through each domain wall. **e-f.** The wave forms of magnetization components of the two magnetic sublattices at a given time.

Supplementary Note 1: Stability of domain wall with DMI

In the presence of DMI, for an antiferromagnetic wire along $\hat{\mathbf{x}}$ direction with uniaxial easy axis in $\hat{\mathbf{z}}$ as shown in Fig. 1, the stable magnetic texture can be either a single domain, a domain wall, a spin spiral, or a mixture of domain wall and spin spiral structure, as described by [1]

$$\left(\frac{\mathrm{d}\theta_0}{\mathrm{d}x}\right)^2 = \frac{C + \sin^2\theta_0}{\varDelta^2} \quad \text{and} \quad \phi_0 = \frac{\pi}{2}, \tag{1}$$

where θ_0, ϕ_0 are the polar and azimuthal angles of the staggered magnetic order $\boldsymbol{n}_0 = (\boldsymbol{m}_1 - \boldsymbol{m}_2)/2$, $\varDelta = \sqrt{A/K}$ is the domain wall width with K and A being the anisotropy and exchange constants, and C is a constant. When $C = 0$, the inhomogeneous solution to Supplementary Equation (1) is a domain wall. For this case, Supplementary Equation (1) does not involve DMI explicitly. The effect of DMI is to determine the domain wall type (Bloch or Néel) and chirality (left- or right-handed), *i.e.* the value of ϕ_0 in Supplementary Equation (1). [1–3] When $C \neq 0$, the solution to Supplementary Equation (1) corresponds to a mixture of domain wall and spin spiral.

The value of C in Supplementary Equation (1) relies on the boundary conditions, and the typical ones include

$$\begin{cases} \text{a). Free:} & \left.\frac{\partial\theta_0}{\partial x}\right|_{x\to\pm\infty} = 0, \\ \text{b). Pinned:} & \theta_0|_{x\to-\infty} = 0, \quad \theta_0|_{x\to+\infty} = \pi, \\ \text{c). DM:} & \left.\frac{\partial\theta_0}{\partial x}\right|_{x\to\pm\infty} = \frac{D}{2A}. \end{cases} \tag{2}$$

The free boundary condition a) means that the exchange torque vanishes at the boundaries; the pinned boundary condition b) means that the magnetization directions at two ends are pinned to $\pm\hat{\mathbf{z}}$; while the the DM boundary condition c) means that the exchange torque and DM torque cancel with each other at the boundaries. All three boundary conditions can be constructed in an antiferromagnetic nanowire. Specifically, the free or pinned boundary can be created by using the exchange bias effect. [4–9]

Ref. 1 studies the DM boundary condition case, and shows that, depending on the strength of DMI in comparison to a threshold value of $D_\mathrm{c} = 4\sqrt{AK}/\pi$, the ground state can be the homogeneous state or a domain wall for $D < D_\mathrm{c}$, and a mixture of domain wall and spin spiral for $D > D_\mathrm{c}$. However, for the free and pinned boundary conditions, we show that, the domain wall state remains stable even for $D > D_\mathrm{c}$, and the domain wall profile is identical to the standard Walker profile as confirmed by micromagnetic simulations shown in Supplementary Figure 1. For $D > D_\mathrm{c}$ and the free boundary condition, we also show in the Supplementary Movie 1 that a prepared spin spiral structure relaxes back to the domain wall structure in micromagnetic simulations.

In this study, the asymmetry in effective potentials for two linear polarizations depends on the strength of DMI, therefore, to maximize the polarizing and retarding effects, it is beneficial to have strong DMI. In the main text, we choose a value of $\tilde{D} \equiv D/\gamma = 3 \times 10^{-3}$ A, which is larger than $\tilde{D}_{\mathrm{c}} = 1.44 \times 10^{-3}$ A. To stabilize a domain wall structure, we use the free boundary condition at the two ends of the nanowire.

Supplementary Note 2: Polarizing and retarding effects at smaller D

The polarizing and retarding effects demonstrated in the main text for relatively large D sustains at smaller DMI strength D. We investigate the polarization-dependent scattering behaviors for a weaker DMI of $\tilde{D} = 1.4 \times 10^{-3}$ A, which is below the threshold $\tilde{D} < \tilde{D}_{\rm c}$, therefore the domain wall configuration is always stable regardless of the boundary condition, as shown in Supplementary Figure 1(b).

Similar to Fig. 2, we plot in Supplementary Figure 2(a-b) the transmission probabilities and the relative phase delays for two different values of D. It can be seen that the polarizing and retarding effects remain for small D, and not surprisingly are less perfect than the large D case shown in the main text. However, the polarizing and retarding effects at smaller D can always be enhanced by chaining two or more domain walls, as shown in the green curves in Supplementary Figure 2(a-b), which corresponds to the case of a double-domain-wall structure with a separation of $335~\mathrm{nm}$. At a working frequency of 8.0 GHz for polarizer, the transmission probability $T_x \approx 0.05 \ll T_y \simeq 1$. In Supplementary Figure 2(a), the transmission probability T_x for the double-domain-wall shows oscillating behavior as function of spin wave frequency, which is due to the multiple reflections between two domain walls, similar to the quantum double potential barrier case. In Supplementary Figure 2(b), the relative phase delay between two linear polarizations is also reduced due to the smaller D. But with two domain walls in series, the phase delay is more or less doubled.

The functionality of the spin wave polarizer at $\tilde{D} = 1.4 \times 10^{-3}$ A is shown by micromagnetic simulations in Supplementary Figure 2(c) with a double-domain-wall structure. At a working frequency of $\omega/2\pi = 8.0$ GHz, when circular spin waves are injected from the left side with equal amplitudes of x- and y-polarization components, the y-component almost passes through the double-domain-wall perfectly, while only a fraction of the x-component is able to transverse the domain walls, identifying the effective polarizing behavior in a double-domain wall.

The functionality of the spin wave retarders at $\tilde{D} = 1.4 \times 10^{-3}$ A is shown by micromagnetic simulations with 4 consecutive domain walls in Supplementary Figure 2(d-f). At working frequency $\omega/2\pi = 14.7$ GHz, the relative phase delay between the x- and y-polarizations across each domain wall is $\delta\varphi_{xy} = \pi/4$, therefore one domain wall is an one-eighth waveplate. As demonstrated in Supplementary Figure 2(d), the relative phase increases by $\pi/4$ when crossing each domain wall. Such a relative phase change across domain wall is also shown by the instantaneous wave forms in Supplementary Figure 2(e-f).

Supplementary Note 3: Green function calculations in the magnetic system

The Green function method used in this paper is based on the procedures described in Ref. 10, which is originally developed and widely used for studies in electronic transport systems, [10–12] but applicable to this magnetic system when properly formulated. For convenience of numerical implementations, equations (2-3) are recast to the discrete form,

$$-i\omega m^{\phi}_{n,\mp} = \tilde{A} m^{\theta}_{n+1,\pm} + \tilde{A} m^{\theta}_{n-1,\pm} - \left[2\tilde{A} + V_{\rm K}(x_n) + J \mp J\right] m^{\theta}_{n,\pm}, \tag{3}$$

$$+i\omega m^{\theta}_{n,\pm} = \tilde{A} m^{\phi}_{n+1,\mp} + \tilde{A} m^{\phi}_{n-1,\mp} - \left[2\tilde{A} + V_{\rm K}(x_n) + J \pm J + V_{\rm D}(x_n)\right] m^{\phi}_{n,\mp}, \tag{4}$$

where ω is the spin wave frequency, n is the lattice index, a is the effective lattice constant, $x_n = na$ is lattice position, $\tilde{A} = A/a^2$ is the exchange constant in lattice model. Supplementary Equations (3)(4) mimic a 4-orbital tight binding model for an electronic transport system. The domain wall here in the magnetic system plays the role of the scattering region in the electronic system, and two domains as the leads. The exchange term $\tilde{A}$ is the hopping term between neighbouring sites, and $V_{\rm K}(x_n), V_{\rm D}(x_n)$ are the on-site potentials.

Similar to the procedures in an electronic system, we start by dividing the magnetic system presented in Supplementary Equations (3)(4) into blocks, with central domain wall composed by blocks 1 to N. In the left/right uniform domain, there are only x- and y- polarized spin wave modes exists as the Bloch eigenmodes, denoted as $\mathbf{u}_{\mathrm{L/R},p}$ with $p = x, y$. To calculate the transmission coefficients $t_{p,p'}$ (which contains both the transmission probability and phase information) between the polarized spin wave modes p and p' across the domain wall, we may directly employ a varied form of the Fisher-Lee relation [10, 13]

$$t_{p,p'}(\omega) = \tilde{\mathbf{u}}^{\dagger}_{\mathrm{R},p}(\omega)\mathbf{G}_{N+1,0}(\omega)[\mathbf{G}^{(0)}_{00}(\omega)]^{-1}\mathbf{u}_{\mathrm{L},p'}(\omega), \tag{5}$$

where $\tilde{\mathbf{u}}_p$ is the duel vector of $\mathbf{u}_p$, $\mathbf{G}_{N+1,0}$ is the Green function between block $N+1$ at the right domain and the block 0 at the left domain of the studied system, and $\mathbf{G}^{(0)}_{00}$ is the Green function between the same block of a infinite system extended from the left uniform domain. Since there is no inter-polarization coupling in Supplementary Equations (3)(4), only the transmission between the same polarization mode $t_{p,p}$ has finite value.

Supplementary References

[1] Rohart, S. & Thiaville, A. Skyrmion confinement in ultrathin film nanostructures in the presence of Dzyaloshinskii-Moriya interaction. *Phys. Rev. B* **88**, 184422 (2013).

[2] Heide, M., Bihlmayer, G. & Blügel, S. Dzyaloshinskii-Moriya interaction accounting for the orientation of magnetic domains in ultrathin films: Fe/W(110). *Phys. Rev. B* **78**, 140403 (2008).

[3] Mulkers, J., Van Waeyenberge, B. & Milošević, M. V. Effects of spatially engineered Dzyaloshinskii-Moriya interaction in ferromagnetic films. *Phys. Rev. B* **95**, 144401 (2017).

[4] Mauri, D., Siegmann, H. C., Bagus, P. S. & Kay, E. Simple model for thin ferromagnetic films exchange coupled to an antiferromagnetic substrate. *J. Appl. Phys.* **62**, 3047–3049 (1987).

[5] Kim, J.-V. & Stamps, R. L. Hysteresis from antiferromagnet domain-wall processes in exchange-biased systems: Magnetic defects and thermal effects. *Phys. Rev. B* **71**, 094405 (2005).

[6] Nolting, F. *et al.* Direct observation of the alignment of ferromagnetic spins by antiferromagnetic spins. *Nature* **405**, 767–769 (2000).

[7] Logan, J. M. *et al.* Antiferromagnetic domain wall engineering in chromium films. *Appl. Phys. Lett.* **100**, 192405 (2012).

[8] Tveten, E. G., Qaiumzadeh, A. & Brataas, A. Antiferromagnetic Domain Wall Motion Induced by Spin Waves. *Phys. Rev. Lett.* **112**, 147204 (2014).

[9] Cheng, R. & Niu, Q. Dynamics of antiferromagnets driven by spin current. *Phys. Rev. B* **89**, 081105 (2014).

[10] Khomyakov, P. A. *et al.* Conductance calculations for quantum wires and interfaces: Mode matching and Green's functions. *Phys. Rev. B* **72**, 035450 (2005).

[11] Ando, T. Quantum point contacts in magnetic fields. *Phys. Rev. B* **44**, 8017–8027 (1991).

[12] Datta, S. *Electronic transport in mesoscopic system*, (Cambridge University Press, 1997).

[13] Fisher, D. S. & Lee, P. A. Relation between conductivity and transmission matrix. *Phys. Rev. B* **23**, 6851–6854 (1981).
